# Reaction to the Solution: Lead Exposure Following Partial Service Line Replacement

**DOI:** 10.1289/ehp.118-a202

**Published:** 2010-05

**Authors:** Rebecca Renner

**Affiliations:** **Rebecca Renner**, PhD, of Williamsport, PA, is a long-time contributor to *EHP* and *Environmental Science & Technology*. Her work has also appeared in *Scientific American*, *Science*, and Salon.com

The safety of a commonly used construction technique for getting the lead out of drinking water—digging up old lead water pipes (“service lines”) and replacing a portion with new copper pipe—has been debated for many years. At best, critics charge, this technique may waste millions of dollars by failing to reduce levels of lead in drinking water. At worst, the partial replacement technique can backfire and substantially increase lead levels for months or longer. The Centers for Disease Control and Prevention (CDC) recently warned public health officials that new agency findings suggest partial replacement of lead service lines may be linked to an increased incidence of high blood lead levels in children. Some drinking water experts are now saying the CDC and the U.S. Environmental Protection Agency (EPA) should consider jointly recommending a moratorium on partial service line replacement based on this new information.

To understand the potential impact of partial lead service line replacement, Virginia Tech environmental engineer Marc Edwards suggests an analogy with lead paint. “We all understand that lead paint becomes a hazard if it’s disturbed,” he says. “Our research indicates that doing a partial [lead] service line replacement is analogous to creating a source of lead dust right in your home, where it is very accessible by children. The ‘dust’ in this case, which is in the form of lead rust on the pipe, builds up continuously over time. If and when it comes off [the pipe], the amount of lead [in drinking water] can vastly exceed health standards.” He adds, “But instead of realizing this is a potential hazard, homeowners have a false sense of security, because they are told that partial service line replacements are being conducted to improve the situation.”

“There is no doubt that partial lead service line replacements can result in significantly elevated levels of lead in tap water and that this contamination can continue for weeks and months, particularly in situations where [drinking water] corrosion control is not optimized,” says EPA chemist Michael Schock. “Why and where these high levels occur is still the topic of research, but their occurrence is fact.”

## One Thing Leads to Another

The EPA started regulating lead in tap water in 1991 in the wake of numerous health studies that linked lead in drinking water to cases of severely elevated blood lead [see “Out of Plumb: When Water Treatment Causes Lead Contamination,” *EHP* 117:A542–A547 (2009) and “Exposure on Tap: Drinking Water as an Overlooked Source of Lead,” *EHP* 118:A68–A74 (2010)]. Water companies generally try to keep lead levels low by controlling water chemistry; water that is too corrosive can liberate more lead from pipes and solder. But if tap water lead levels continue to exceed the action level of 15 ppb after corrosion control is implemented, the federal Lead and Copper Rule (LCR) requires water utility companies to begin replacing lead service lines.

Initially the LCR required the replacement of the entire lead pipe, both the publicly and privately owned sections. But requiring water utilities to remove privately owned lead service lines raised constitutional and legal issues in terms of private property and eminent domain. A 1994 challenge in the DC Circuit Court by the American Water Works Association (AWWA) limited the EPA’s jurisdiction to just the public portion of the service line. As a result the LCR was revised in 2000 to allow for partial service line replacement, although utilities may offer homeowners the option of replacing their portion of the line at the homeowner’s cost.

This change was backed by the EPA’s interpretation of field studies that examined the impact of partial replacement, according to EPA spokeswoman Enesta Jones. She explains that the limited number of available studies all eventually found that lead levels declined after the new pipes were installed.

Jeff Kempic, treatment technology and cost team leader with the EPA Office of Ground Water and Drinking Water, notes that weighing the relative benefits or disadvantages associated with partial service line replacement is a task that extends beyond the EPA’s control. “The vast majority of partial lead service line replacements occur on a voluntary basis and are not covered by the LCR requirements,” he says.

In the United States lead service lines are most prevalent in the older cities of the Northeast and Midwest. Their installation was actually required by plumbing codes in U.S. cities through the 1950s, and in some places they were installed up until Congress imposed restrictions on the lead content of plumbing in 1986. The 1990 report *Lead Service Line Replacement: A Benefit-to-Cost Analysis*, prepared for the AWWA, provides the most recent estimates for the amount of lead plumbing in the United States: approximately 3.3 million lead service lines and 6.4 million lead connections (curved pieces that join one pipe to another). Today, there could still be millions of U.S. homes with lead service lines.

Although numbers vary dramatically from city to city or even from home to home, a national survey conducted as part of a Water Research Foundation (WaterRF) project and published in the 2008 report *Contribution of Service Line and Plumbing Fixtures to Lead and Copper Rule Compliance Issues* found the average total length of service lines was 55–68 feet, with 25–27 feet of that length controlled by the water utility. The cost of replacing the customer portion can add up to several thousand dollars, and few customers do so voluntarily, according to the AWWA.

Providence Water in Rhode Island is currently required to replace 7% of its 26,000 lead service lines each year to comply with the LCR. Water companies also may voluntarily replace large numbers of lead service lines as a proactive measure to ensure water quality. Louisville, Kentucky, is replacing lead service lines voluntarily at an annual cost of $1.5–2 million, according to 2008 figures from the Louisville Water Company.

## Partial Replacement and Elevated Water Lead

But Washington, DC abandoned an extensive and expensive lead service line replacement program in 2008 in part due to data indicating partial replacement caused higher levels of lead in drinking water for at least several months, according to George Hawkins, general manager of the District of Columbia Water and Sewer Authority, Washington, DC’s water utility. The district now does partial service line replacements only when road repair or a broken water main necessitates such work, says Hawkins. In these cases the water company provides filters and monitors homeowners’ drinking water for 5 months.

A 1992 report for the UK Department of the Environment titled *Economics of Lead Pipe Replacement* noted that in theory, when a lead pipe is partially replaced, the amount of lead in water should be reduced because there is less expanse of lead in contact with the water. But practical experience “shows an unexpected rise in measured lead levels after the replacement has been carried out,” stated the report. “The estimates for the duration of the observed rise vary from 4 up to 18 months.”

One explanation for the spike is that the replacement work disturbs the pipes and knocks off lead-bearing pipe scale (built-up minerals that coat the inside of the pipe). In addition, “partial replacements using copper piping can result in the creation of a galvanic cell,” states the UK report. “This chemical cell can exacerbate the problem of plumbosolvency [lead release] and give rise to increased and erratic levels of lead observed at the tap. The effect can be persistent and may well annul any beneficial effects of reducing the length of lead pipe in the system.”

Galvanic corrosion is an electrochemical process in which a metal corrodes when it is in contact with a different metal and both are immersed in an electrolyte. Galvanic corrosion creates the voltage in car batteries. This potentially powerful electrochemical phenomenon is also used to extend the life of steel water heaters through the use of sacrificial anode rods, so named because they are designed to dissolve and thereby protect other metallic parts; the sacrificial rod corrodes, not the water tank.

When copper water pipe is connected to lead water pipe, standard electrochemistry indicates the lead pipe should be more susceptible to galvanic corrosion. If corrosion is significant and long-lasting, it would significantly add to lead release. “The possible role of galvanic corrosion in the release of lead to water was recognized over 150 years ago, although we don’t fully understand the conditions that promote or deter it,” says Simoni Triantafyllidou, a graduate student in Edwards’ lab at Virginia Tech.

In volume 35, issue 5 (1981) of the *Journal of the Institution of Water Engineers and Scientists*, A. Britton and W.N. Richards described a number of examples taken from Scotland, where high prevalence of lead water pipes, lead water tanks, and naturally corrosive water caused long-standing problems with high levels of lead in drinking water. They studied 195 households in Glasgow, 186 of which had some lead water pipes. The 69 households with mixed lead and copper piping were more likely to have high levels of lead in their tap water. “Occasionally the insertion of copper pipe can produce particularly bad results and despite satisfactory pH control it may be impossible to obtain any satisfactory samples,” they wrote. Ironically, this is one of the studies used by the EPA to support partial service line replacements, notes Edwards, who adds that other researchers in England, most prominently Oliphant and Gregory, noted that in many waters serious lead contamination was caused and influenced by galvanic corrosion.

More recently, motivated by elevated levels of drinking water in Greenville and Durham, North Carolina, which appear to be related to water treatment changes that increased the ratio of chloride to sulfate in drinking water, Edwards and Triantafyllidou have been conducting experiments that evaluate galvanic corrosion by measuring electrical current between joined copper and lead pipes and also by measuring release of dissolved and particulate lead. “We have confirmed that a high chloride-to-sulfate mass ratio can trigger galvanic corrosion of lead solder and release hazardous levels of lead into drinking water, consistent with early English studies,” says Triantafyllidou. “We believe this explains the elevated levels of lead in Greenville and Durham.”

The chloride-to-sulfate mass ratio can change in treated drinking water due to a change in coagulant chemicals used to remove organic matter, as occurred prior to spikes in water lead in Greenville and Durham. Other factors that could increase this ratio include road salt entering a water supply from runoff, desalination, anion exchange treatment, or brine from sodium hypochlorite generators (used to disinfect drinking water) leaking into a water supply.

In contrast to Edwards and Triantafyllidou’s galvanic corrosion study, which was funded by WaterRF, another WaterRF-funded experimental study conducted by consultancy HDR and published in March 2010 found that lead release due to galvanic corrosion was short-lived and transient. “Both projects recommended that more research be done in this area to better understand the short- and long-term effects of galvanic corrosion, as well as water quality effects that both exacerbate and mitigate galvanic corrosion,” says WaterRF project manager Traci Case. “For now, we know that galvanic corrosion leads to lead release after partial [replacements].”

Schock and Michael DeSantis, a mineralogist with EPA contractor Pegasus Technical Services, have been examining old connections between lead and copper or lead and brass to look for signs of galvanic corrosion. Their preliminary examination of more than a dozen decades-old pipes was presented at the AWWA Water Quality Technology Conference in November 2009 and has revealed clear examples of galvanic corrosion. “We see corrosion very locally at lead junctions,” says Schock. “The minerals formed on some of the lead sides of the connections show that locally the lead can be hundreds of milligrams per liter, but we can’t tell how long it persists or how much of the high lead generated there [gets] into the water.” But they have also found pipes where corrosion is minor as well as joints where the lead appears stable and the copper has corroded.

“There are many waters in which we are sure that galvanic corrosion is not a problem,” says Edwards. “But our lab data show that the worst case can be quite bad. When problems occur, they can be very hard to detect due to erratic release of lead scale at the [lead–copper] joint. More research is needed to understand the issues associated with sloughing off of lead scale and galvanic corrosion,” he says.

## Signs of Health Effects

In January 2010 the CDC announced the findings of an unpublished epidemiologic study suggesting a relationship between elevated blood lead levels in children and partial lead service line replacements. A notice published on the agency’s website (http://www.cdc.gov/nceh/lead/waterlines.htm) advises public health managers that customers should be informed when partial lead service line replacement occurs so they can take steps such as flushing taps and cleaning aerators after service line disruption. The CDC notice does not address how long after the service line replacement taps should be flushed or how many minutes the tap should be flushed at each use before using the water.

The notice, written by Howard Frumkin, CDC’s former director of the National Center for Environmental Health, says the study’s preliminary results suggest that when the public portion of a lead service line is replaced, children are more likely to have blood lead levels of 10 μg/dL or higher, compared with children living in housing with undisturbed lead service lines or nonlead service lines. The epidemiologic study of children living in Washington, DC, is undergoing peer review, and publication in a scientific journal is anticipated.

“It is important to share these preliminary findings with the nation’s childhood lead poisoning prevention managers to provide guidance about lead-safe water practices in homes with lead-based water lines or lead solder following plumbing work including water service line replacement,” says CDC spokeswoman Bernadette Burden in explaining why the agency issued the notice in advance of the paper’s publication.

“There have always been doubts about the benefits of partial service line replacement,” says Alan Roberson, director of security and regulatory affairs for the AWWA. “The CDC study is the first I know of that links partial replacement to adverse health effects, but these new findings appear to confirm the existing, long-standing doubts.”

Jim Elder, who headed the EPA drinking water program from 1991 to 1995, says, “Given these data, CDC and EPA should jointly recommend a moratorium on partial service line replacements.”

Roberson agrees that a moratorium makes sense. Following an October 2008 EPA meeting that discussed possible long-term revisions to the LCR, AWWA representatives submitted comments to the agency stating that “aggressive and expensive [lead service line] replacement programs which do not result in exposure reductions and may in fact result in exposure increases over the short-term seem to be an inappropriate allocation of limited funds. The primary role of [lead service line] replacement in the LCR appears to be as a regulatory ‘hammer,’ and based on available research, it appears to be a hammer with previously unanticipated risks that are inappropriate for the LCR.”

The EPA has committed to re-evaluating its regulations that cover partial lead service line replacement. In testimony before the Senate Environment and Public Works Committee in late 2009, EPA Assistant Administrator for Water Peter Silva committed to finalizing long-term revisions to the LCR by 2012. “But to meet that timetable with meaningful proposals, EPA should be doing research and fact-finding to sum up the current situation and look into options,” says Roberson. “There are no indications as far as I’m aware that they are doing this.”

## Possible Solutions

Everyone contacted by *EHP* for this story, including EPA sources, agrees that full lead service line replacement is preferable to partial replacement. However most believe the financial and legal impediments to full replacement are difficult to surmount. When partial lead service line replacements occur, the tap should be flushed; utility recommendations vary from 15 minutes to 1 hour of full-flow flushing immedately after partial service line replacement. Afterwards, residents should use “point-of-use” home water filters that are certified under National Sanitation Foundation/American National Standards Institute (NSF/ANSI) Standard 53 for both particulate and dissolved lead (or NSF/ANSI Standard 58 for reverse osmosis systems), according to Schock. Kempic says one of the long-term LCR revisions currently under consideration at the EPA is a requirement that water filters be supplied to homes where partial service line replacement occurs.

Since galvanic corrosion requires contact between metals, there may be a fairly simple engineering solution—the use of a longer brass connector might put enough distance between the lead and copper to reduce the corrosion, says Edwards. However, the effectiveness of this solution would depend on the composition of the brass alloy used and the pH of the water. Edwards also says the use of some sort of nonconducting dielectric might be beneficial. Existing pipes can also be lined with epoxy resin, although such lining has not been tried on a large scale in the United States.

In the United Kingdom, where approximately 40% of homes have lead pipes, partial service line replacement is not widespread, according to corrosion expert Colin Hayes of Swansea University. “The emphasis in the UK has been on the optimization of corrective water treatment, most of which includes dosing orthophosphate,” Hayes says. He says the success of corrective water treatment has much reduced the need for lead pipe replacement in the short term; in England and Wales, 99.77% of samples in 2008 complied with the current European lead standard.

Hayes says UK systems use relatively high levels of orthophosphate—about 3 times the levels used in the United States. However, in the United States, concerns about increasing levels of phosphate loading to the environment might limit this option. Orthophosphate dosing has not been implicated in such problems in the United Kingdom, Hayes says, although he acknowledges that “elsewhere in Europe there have been environmental concerns about using orthophosphate—mostly unfounded—and many water companies have been replacing their lead connection pipes but not those lead pipes owned by consumers.”

A potential legal solution might be to stop partial service line replacements and instead require full replacement when property changes hands. Analogous steps are currently taken to correct problems with underground fuel oil tanks in some states such as Washington.

“Taken as a whole, the data suggest that galvanic corrosion can occur, but we don’t know enough about the specific conditions, the scale on which it occurs, or what causes worst-case effects,” says Schock. “Having said that, it is hard to see how any public agency could justify to the public purposely leaving even part of the lead pipe in the ground. Advising people to use a filter and flush their taps following partial replacement should help. This issue definitely cuts across public health, scientific, technical, and legal issues. It would be great to see collaboration among these disciplines and across agencies to figure this out.”

## Galvanic Corrosion

When lead and copper plumbing pipes are connected, a galvanic cell is created. Metallic lead serves as the anode and is oxidized (corroded). The copper pipe serves as the cathode, and the drinking water flowing through the joined pipes serves as the electrolyte.

Brass contains varying percentages of copper, zinc, lead, and other metals. Some brass alloys also can be cathodic to lead; the makeup of the given alloy determines if the brass or the lead will corrode, and the pH chemistry of the water also makes a difference. In some waters, use of a short brass connector may alleviate the very high corrosion rate that can occur when lead is connected directly to copper. However, that is not always the case—as seen in these photographs of a curved brass “gooseneck” soldered to a piece of lead pipe that has corroded badly.

## Timing Is Everything

A 2005 conference proceeding document prepared by John Joseph Wujek for the District of Columbia Water and Sewer Authority, Minimizing Peak Lead Concentrations after Pwartial Lead Service Replacements, has often been cited as showing how partial lead service line replacement can improve water quality if the replacement is followed by vigorously flushing water through the tap. However, this study suffers from a flaw, according to EPA Region 3 environmental scientist Jennie Saxe.

In Washington, DC, high levels of lead occurred in part due to a November 2000 change in the disinfectant used to treat water from chlorine to chloramine. In the spring of 2004, the city switched back to chlorine for a month to control bacteria, and lead levels plummeted citywide. For the project described by Wujek, the initial, pre-replacement measurements of lead in water were made close to the time when chloramine was in the water, and lead levels were high. The post-replacement sampling was impacted by benefits from chlorine in the water when lead levels were low.

The factors that marred this study illustrate how difficult it can be to conduct field studies on the effects of partial service line replacement on tap water, says Edwards. “Water che mistry, temperature, whether the pipes have been flushed, even how fast the tap is running when samples are collected—all these factors affect field study results,” he says. “With no standard protocol, these studies are difficult to compare and may provide misleading results.”

## One City’s Experience with Full Lead Service Line Replacement

Although there is general agreement that replacing the entire lead service line is preferable to partial service line replacement, few utilities in the United States or elsewhere have found ways to conquer the legal and financial hurdles that block full replacement. One city that has succeeded is Madison, Wisconsin.

In 1994 Madison Water Utility was faced with drinking water lead levels that exceeded EPA requirements, but studies found that most standard chemical corrosion treatments were ineffective at reducing lead levels. The exception was orthophosphate; however, orthophosphate dosing was unpalatable because the area’s wastewater treatment plant had just built a biological removal system for phosphate to manage nutrient loading to the Great Lakes. In a 2006 case study, consulting engineer Abigail Cantor of Process Research Solutions wrote, “If phosphorus was to be added to the drinking water, the removal system would not work properly and a chemical phosphorus removal system would need to be added. In addition, the water that would runoff directly to the lakes would carry phosphorus with it.” Already, she observed, there was talk in the city council of banning phosphorus lawn fertilizers in Madison.

Madison determined that the long-term costs of orthophosphate would be greater than the cost of total lead service line replacement and that replacing lead service lines would reduce lead levels more than dosing with orthophosphate would. The state regulator, the Wisconsin Department of Natural Resources, agreed that removing lead service lines was the only reasonable means of corrosion control available—but this meant removing the entire lead service line, both the public and private portion.

When the program began on 1 January 2001, there were approximately 6,000 lead service lines on the publicly owned portion and 5,000 on the homeowner side. The state set a goal of replacing all service lines by 2011. In 2000 the city passed an ordinance that prioritized replacements in schools and day care facilities.

But there was disagreement about who should pay for replacing privately owned service lines, says utility finance manager Robin Piper. First the utility tried to add a charge to the bills of its more than 60,000 customers, but this was vetoed by Wisconsin’s utilities commissioners. “The sewer authority was able to add a surcharge, and this makes sense because choosing service line replacements over orthophosphate dosing saved water treatment costs,” he says. Additionally monies came from cell phones—the utility receives rent for allowing cell phone antennas to sit atop its water towers. “At the height of replacement efforts we were spending $500,000 to $600,000, and the antenna rental fees contributed several hundred thousand,” Piper explains.

The city uses this money to help reimburse customers for half the cost of replacing their lead service line, up to $1,000. For low-income customers, the city provides a loan for the other half of the cost, with repayment deferred until the property is sold. Costs for the utility portion have averaged about $2,000, and for the customer portion about $1,400, Piper says. To date Madison is on target with 5,394 pipes replaced.

## Figures and Tables

**Figure:**
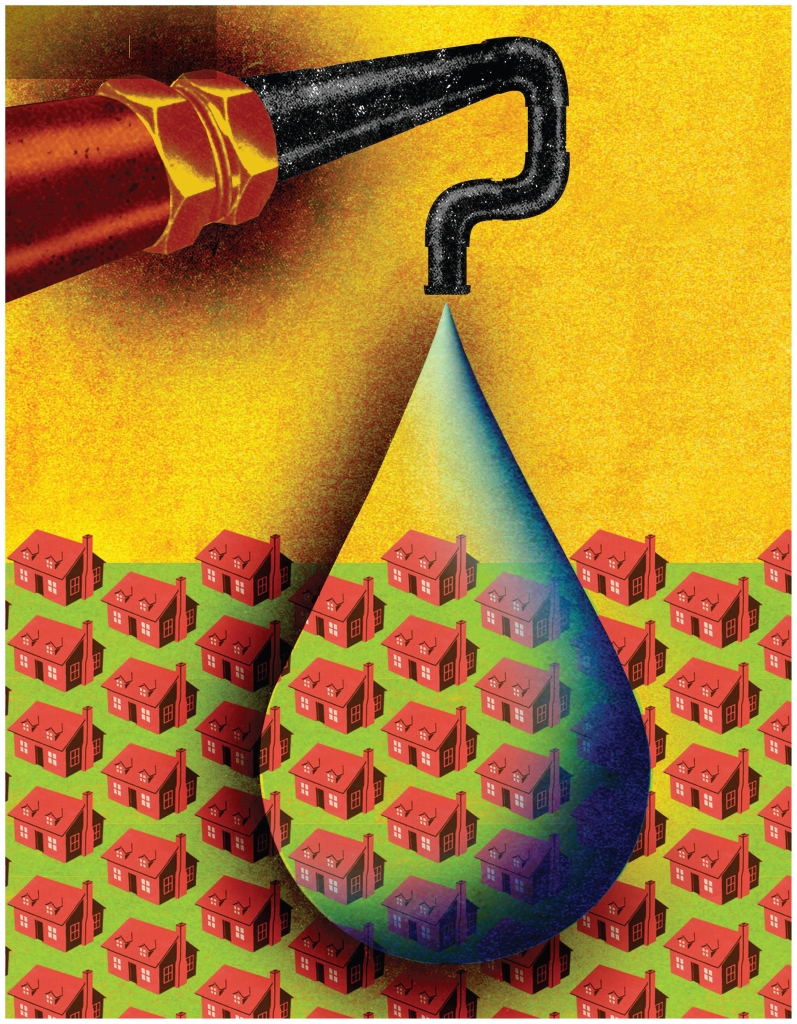


**Figure:**
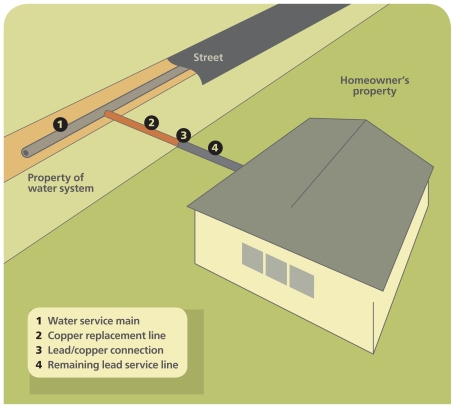
Initially the Lead and Copper Rule required water utilities to replace entire lead service lines, but constitutional and legal concerns about this stipulation led to a revision of the law in 2000 to allow for partial service line replacement. Utilities may offer homeowners the option of replacing the privately owned portion of the line at the homeowner’s cost, but few exercise this option.

**Figure:**
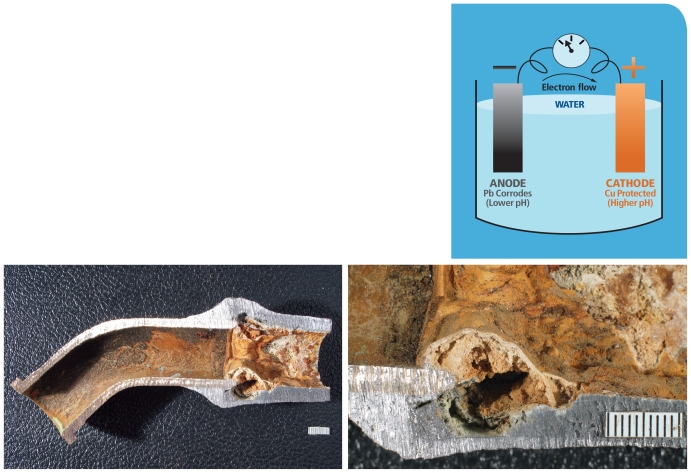


**Figure:**
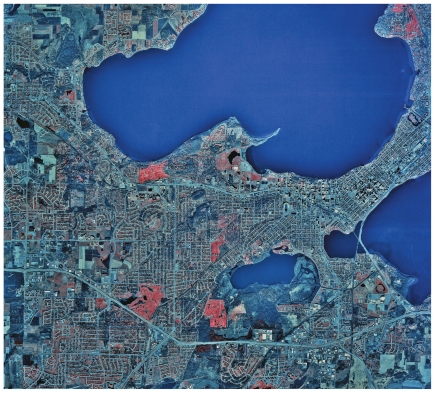
The threat of lake nutrient loading made orthophosphate a no-go for Madison, Wisconsin.

